# That Resection

**Published:** 1895-09

**Authors:** A. M. Denman

**Affiliations:** Lufkin, Texas


### Correspondence.

### That Resection.

LETTER FROM DR. A. N. DENMAN.

LUFKIN, TEXAS, August 20, 1895.

*Editor Texas Medical Journal:*

The article in the July number of your excellent journal, written by Dr. J. R. Stuart, of Houston, and entitled ''Ununited Fractures,'' does me such rank and manifest injustice, and so completely misrepresents me and misstates the case, that I feel compelled, much against my inclination, to notice it. The article is a striking illustration of what Shakespeare calls ''damning with faint praise.'' In one line the writer would have you believe that I am a ''most excellent surgeon,'' and in the next makes me appear to any reader of good common sense a most consummate fool. I must, therefore, crave space in the JOURNAL for a reply, in which, while trying to set myself right before the readers of the JOURNAL, I shall not attempt to detract anything from the glory which the writer feels has settled on his head in consequence of his brilliant (?) exploit.

I will state the case as it occurred, and confining myself to facts, will not let my fancy or an excited imagination paint it, as did your correspondent in his vain-glorious and quixotic account.

On October 11, 1894, one Chas. Williams, an employe of the H. E. & W. T. R'y, while attempting to couple cars, was knocked down by protruding lumber and one wheel of the car ran over the lower third of thigh, inflicting a compound comminuted fracture, making a very large opening, six and one-half inches long, torn in every direction, detaching muscle, ligaments and bone; in fact, crushing and bruising nearly everything with which it came in contact, the blood vessels only escaping. I thorougly cleansed the negro from head to foot (he was very dirty), removed all the detached bones, which was four and a half or five inches of the entire thickness of femur, tied all bleeding vessels, approximated all detached muscles and tendons and securely sutured them with sterilized silk-worm gut, removed everything of an offending nature, put the leg in an extension aseptic and antiseptic dressing, with a hope of getting *union at full length*, thereby giving the negro the best chance for a useful limb. It would have taken at least five or six months for sufficient callus and new bone formation to have effected the desired union, therefore the bones were not approximated at all, as Dr. Stuart would have you believe; in fact, the title of his paper is purely a misnomer, as there was a space to be filled of four or five inches, and no one would expect union, if at all, under five or six months. There was no chance for union, and it was not my intention for fractured ends to unite, until sufficient new bone had formed to fill up the gap or hiatus left by the removal of the splintered shaft, which, I thought, if properly handled, with modern antiseptic precaution, might be possible. I had seen bone reform, as no doubt every surgeon of experience has, in cases where a considerable portion has been destroyed or removed, and make useful members. Knowing this, and believing that such a thing was possible in this case, in justice to my patient I felt it my duty to give him the chance; knowing that, in case of failure, after sufficient time, I then could remove splintered ends, if necessary, approximate bones, and get union by *shortening;* this I wished to avoid, and I believe, had the case not been meddled with, it would have been accomplished.

I placed my patient in a well ventilated room after thoroughly cleaning and fumigating it, placed him in charge of a faithful nurse. I kept a close watch over him, redressed the wound on the fourth day, found it in a nice healthy condition, after which I dressed the wound every other day until union of all the soft parts had taken place, which was on the sixty-second day from date of injury, without one drop of pus, without one moment's pain, and without any elevation of temperature after the first twenty-four hours; reaction from shock. I kept the leg extended at full length all the time *to prevent union*, observing from time to time the process of bone repair. On the sixty-fourth day, finding sufficient callus and new formed bone to justify (as the leg would remain at full length without the aid of splints), I adjusted the entire limb in a plaster paris dressing, expecting to allow it to remain in place for three months at least. In the meantime I wrote the manager of the H. E. & W. T. Ry. that the man was in fine physical condition, explaining to him the purpose of my treatment, telling him the length of time it might take to effect a cure, and to lessen expense, I suggested it would be best to remove the patient to the hospital, where he could await results.

Dr. Boyles, of the firm of Stuart & Boyles, the chief surgeons of the railroad, came to Lufkin for my patient, and while here I explained to him fully my views, the ends I sought, and the reasons for so doing. In a few days after the arrival of my patient at the hospital, I received a letter from Drs. Stuart & Boyles, stating that they had *approximated the bones;* that I was *mistaken* as to the amount of bone I had removed, that I had left the posterior half of the fractured section, and that they had removed it, and *hoped to get union.* Having the entire four and one-half inches bone above referred to, before me, I felt sure of one of three things; first, that these surgeons had removed the new formed bone; second, that they had not removed any bone at all, or else Chas. Williams had a bone and a half in his thigh; third, that some one of us was mistaken.

As to myself, I am positive that I removed four and one-half inches of bone, involving the entire thickness of femur. It requires no "integrity," on my part, as I still have the bones; besides, about ten or fifteen of our best citizens saw them immediately after their removal. I also exhibited the bones to Drs. Stuart and Red while in the infirmary at Houston. Dr. Red placed the bones together, and stated that the entire thickness of femur had been removed; he also stated that the bone they had removed must have been a *new bone.* If it was anything, it must have been a new formed bone, as Dr. Stuart tells you that when he removed the dressing and made the incision he found the bones over lapping. Now is it reasonable to suppose the bones could over-lap when they were at least four and one-half inches apart, and no shortening in the limb? No man who had a modicum of surgical knowledge would believe such a statement; yet Dr. Stuart would have you believe, as the title of his paper indicates, that we only had a compound fracture to deal with. He is careful not to tell you that the leg was as long as its fellow; or does he tell you about the posterior half of bone which he claims to have removed? No; but calls it ''over lap;'' hence my reason for calling it a misnomer.

I stated at the outset that Dr. Stuart had misrepresented my case from beginning to end. I will modify that statement somewhat. He did give the correct name of the railroad on which the accident occurred; the correct date, and name of party wounded; and possibly his calculation as to weight of car is correct,—and further, suppose he took all the precautions stated by himself about the preparation for his operation at the Infirmary; but it looks strange, if such precautions were taken, to have seen Chas. Williams, whom I had sent there, a short time before, strong and healthy and with normal temperature, looking so lank and lean, and with a temperature ranging from 101 to 105, and *pus pouring out* of such a *carefully dressed* (*?*) wound. It was possibly due to the fact that Dr. Stuart only ''changed his bed *once* a week."

While visiting my patient, in his new quarters at the Infirmary, I saw several other wounded men, and plenty of pus; in fact, pus seemed to be far more plentiful than surgical dressing; possibly it was the old fashioned ''laudable pus,'' which used to gladden the hearts of our honored forefathers; nevertheless, it was *pus*.

The foregoing may all seem harsh, and out of place; but if you who read my reply knew what I do, and had been intimidated as I have been by these great railroad surgeons, you would not think so. They have tried to intimidate me all the way through, in all our correspondence. They have not paid my bill, nor the bills of any one concerned in this case. The cause of intimidation, therefore, I leave readers to infer, as well as the publication of young Stuart's paper, as he sets out by stating three reasons for writing it: First, ''from a professional standpoint;'' second, ''from a legal standpoint,'' and, third, ''from a pecuniary point of view.'' ''Professionally,'' he says, "our pride is wounded." "Legally," we are "liable to be sued for malpractice." "Pecuniarily," "patients *object* to *paying* physicians for such work." His article, you see does me great wrong, in that it reflects seriously upon me; indirectly makes a charge of malpractice against me, and thus seemingly a pretext for refusing to pay my bill. I feel outraged and indignant, and, I think, with cause.

Now, if this teacher of surgery is so instructive, from a "professional, legal and financial point of view," about "ununited fractures," why does he not go to work and fumigate, renovate and scrub with his "solutions of coros. sub. permanganate, pot. alcohol, etc., and clean up those wards and prevent pus? He is careful, he says, to chop the nails off, and throw all the "finger rings" out, but the pus-patients were in evidence during my visit. This he should do, before he attempts to criticise the work of others, or pose as a teacher on "ununited fractures."

Now, what in the thunder is the use for a surgeon to scrub the life out of his patient and himself to get them clean for an operation, and after it is all neatly and nicely done, chuck him in a ward between men who seem to be laid out on purpose for pus culture? and reeking with it. (Laudable pus, I guess.)

It seems to me that Dr. Stuart ought to write an article on "pus, from a professional, legal and pecuniary point of view." I think "our pride" would be far more "wounded," on removing any dressing, to find pus than to find an ununited fracture; that we would be far more "liable to be sued," and still further, "not to be paid"; because a filthy man is to be dreaded; besides it can be established that pus is the result of pure *carelessness* and a *want of skill*, while in the other event, it is not the surgeon's fault, every time.

Dr. Stuart states that I dressed the wound first, and afterwards removed four or five inches of bone. I removed the bones at the first dressing, as stated, before they were all detached, and I removed them without the aid of instruments.

I am very sorry that I am forced to reply to the doctor's article, but I feel it my duty to do so, and trust no one will think me unduly harsh or unethical. I have not written this to instruct any one, as I have done nothing new nor smart, but to vindicate myself from misrepresentation and tacit charge of ignorance and malpractice.

If Dr. Stuart had let my patient alone, I have no doubt he would have recovered with a good leg, not shortened, and I

would have reported the case whether successful or not; but alas, see the result.

I regret that I have neither war record, war stories nor ancestral instruments of hoopskirt origin to relate, with which to round off my paper,—so I will stop, and leave the readers of the JOURNAL to judge between these great surgeons and a plain country practitioner who, nevertheless, has been to school.

A. M. DENMAN, M. D.

